# Plin5 inhibits proliferation and migration of vascular smooth muscle cell through interacting with PGC-1α following vascular injury

**DOI:** 10.1080/21655979.2022.2065762

**Published:** 2022-04-26

**Authors:** Xueqing Gan, Jiaqi Zhao, Yingmei Chen, Yong Li, Bing Xuan, Min Gu, Feifei Feng, Yongjian Yang, Dachun Yang, Xiongshan Sun

**Affiliations:** a Department of Cardiology, The General Hospital of Western Theater Command, Chengdu, Sichuan, China; b Department of Cardiology, The People’s Hospital of Chaotian District in Guangyuan, Guangyuan, Sichuan, China

**Keywords:** Plin5, PGC-1α, VSMC, ROS, neointima hyperplasia

## Abstract

Abnormal proliferation and migration of vascular smooth muscle cell (VSMC) is a hallmark of vascular neointima hyperplasia. Perilipin 5 (Plin5), a regulator of lipid metabolism, is also confirmed to be involved in vascular disorders, such as microvascular endothelial dysfunction and atherosclerosis. To investigate the regulation and function of plin5 in the phenotypic alteration of VSMC, -an animal model of vascular intima hyperplasia was established in C57BL/6 J and *Plin5* knockdown *(Plin5*^±^) mice by wire injure. Immunohistochemical staining was used to analyze neointima hyperplasia in artery. Ki-67, dihydroethidium immunofluorescence staining and wound healing assay were used to measure proliferation, reactive oxygen species (ROS) generation and migration of VSMC, respectively. Plin5 was downregulated in artery subjected to vascular injury and in VSMC subjected to platelet-derived growth factor (PDGF)-BB. Plin5 knockdown led to accelerated neointima hyperplasia, excessive proliferation and migration of VSMC after injury. In vitro, we observed increased ROS content in VSMC isolated from *Plin5*^±^ mice. Antioxidative N-acetylcysteine (NAC) inhibited VSMC proliferation and migration induced by PDGF-BB or plin5 knockdown. More importantly, plin5-peroxlsome proliferator-activated receptor-γ coactivator (PGC)-1α interaction was also attenuated in VSMC after knockdown of plin5. Overexpression of PGC-1α suppressed PDGF-BB-induced ROS generation, proliferation, and migration in VSMC isolated from *Plin5*^±^ mice. These data suggest that plin5 serves as a potent regulator of VSMC proliferation, migration, and neointima hyperplasia by interacting with PGC-1α and affecting ROS generation.

## Graphical abstract

**Figure:**
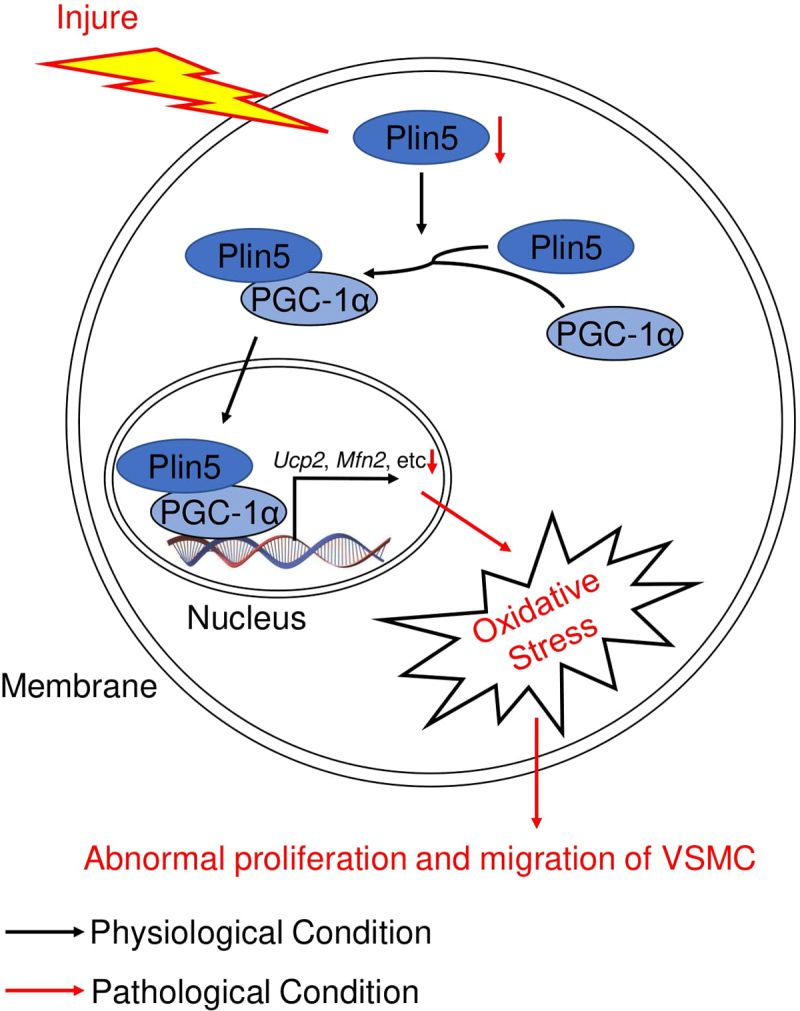

## Highlights

Plin5 is downregulated in PDGF-BB-challenged VSMC and injured artery.

Inhibition of plin5 promotes the proliferation, migration of VSMC and neointima hyperplasia after vascular injury.

Plin5 inhibits VSMC phenotypic transition in a PGC-1α-dependent manner.

## Introduction

Cardiovascular diseases (CVDs) are the major cause of disability and death all over the world. Percutaneous vascular intervention is an important therapeutic method for CVDs, including atherosclerosis, coronary heart disease, and peripheral vascular diseases. However, a proportion of patients develop postoperative vascular restenosis, which largely weakens the curative effect of intervention [1]. Restenosis is a complex and multivariate process. Firstly, stent implantation or angioplasty causes endothelial dysfunction owing to mechanical damage (also called de-endothelization) [2]. Several cytokines and growth factors such as platelet-derived growth factor (PDGF) are then secreted and inflammatory cells (macrophages, neutrophils, etc.) are recruited to the damage site [2]. Simultaneously, vascular smooth muscle cell (VSMC) undergoes a phenotypic switch from quiescent to proliferative phenotype [3]. In addition, the deposition of collagen and extracellular matrix are also involved in the development of restenosis [1]. Revealing the underlying mechanisms of these pathological processes is important for preventing vascular restenosis.

Serving as the major component of vessel, VSMC undergoes a phenotypic transition in response to certain cytokines or growth factors [4]. This transition of VSMC is a crucial part during the development of neointima hyperplasia and vascular restenosis. Quiescent VSMC is well differentiated, contractile and has low levels of proliferation and migration, while proliferative VSMC is dedifferentiated and has strong ability of proliferation and migration [4]. Accordingly, we previously also observed excessive proliferation and migration of VSMC in diseased artery after mechanical damage [5]. Multiple signaling pathways have been proved to be involved in VSMC proliferation and migration. These pathways include mitogen activated protein kinases and phosphatidylinositol-3-kinase/serine threonine protein kinase, etc [6]. Although the application of drug-eluting stents based on above-mentioned pathways has indeed improved the complications of percutaneous inventions, the incidence of restenosis still reaches to 10% [7]. Therefore, searching the novel mechanism that modulates VSMC phenotypic transition is of great significance.

Belonging to the perilipin (plin) family, plin5 is predominantly considered to regulate lipolysis of lipid droplet, fat oxidation, and insulin resistance [8]. Plin5 is highly expressed in tissues and organs that have strong capacity of fat oxidation, including brown adipose tissue, skeletal muscle, and heart [9–11]. Plin5 exerts varied roles in cardiovascular diseases. Overexpression of plin5 inhibits cardiac lipolysis and maintains normal heart function and life span [12], while plin5 knockdown causes reduced substrate availability, cardiac dysfunction, and elevated mortality following myocardial ischemia [13]. Plin5 is also involved in the modulation of vascular diseases. Deletion of plin5 accelerates atherosclerosis via enhancing inflammation, apoptosis, and oxidative stress [14]. Our lab has recently found that plin5 preserves microvascular endothelial cell survival and microvascular integrity in diabetic mice through regulating fatty acid metabolism [15]. However, it is unclear whether plin5 exerts a role in VSMC phenotypic transition and vascular intima hyperplasia.

Elevated reactive oxygen species (ROS) is known to induce oxidative stress and abnormal proliferation and migration of VSMC [16]. We previously found that plin5 deficiency led to enhanced ROS generation in cardiac microvascular endothelial cells [15]. Whether plin5 regulates oxidative stress in VSMC remains unclear. Peroxisome proliferator-activated receptor γ coactivator-1α (PGC-1α) inhibits VSMC proliferation and migration via suppressing oxidative stress [17]. However, the role of PGC-1α in plin5-mediated regulation of VSMC proliferation and migration is unknown.

We hypothesized that plin5 might be a novel molecular mechanism that is involved in inhibiting VSMC phenotypic transition and neointima hyperplasia after vascular injury. Therefore, we used mice whose *Plin5* were knocked down (*Plin5*^±^) to investigate our hypothesis. Our study was expected to provide clues for searching new therapeutic targets of vascular restenosis.

## Materials and methods

### Animals

Wild-type (WT) C57BL/6 J mice were obtained from Dashuo Animal Science and Technology (Chengdu, Sichuan, China). *Plin5*^±^ mice were obtained from Graduate School of Life Science (Hyogo, Honshu, Japan). Male mice (10-week-old) were housed in the following conditions: 12 h (h) regular light/dark cycle, 22–25°C and periodic air changes. Mice have free access to food and water. All experimental procedures were approved by the Institutional Animal Care and Use Committee and the Ethic Committee of The General Hospital of Western Theater Command (No. 2020ky014; Chengdu, Sichuan, China). Injury model of common carotid artery was established as described in our previous literature [18]. Briefly, after anaesthetization with pentobarbital (40 mg/kg, intraperitoneal), a small midline incision in the neck area was conducted. The left common carotid artery proximal to the aortic arch and the left internal carotid artery of the mouse were temporarily blocked. Then, wall of the left external carotid artery was rubbed back-and-forth by a curved, polished guidewire to denude the endothelial cells. The left external carotid artery was occluded proximal to the incision site and the clamps were removed. The incision of neck was finally closed. Mice were anesthetized with pentobarbital (100 mg/kg) and followed by decapitation for execution. Common carotid arteries were harvested 7, 14 and 28 days after injury.

### Cells

VSMCs were isolated from WT and *Plin5*^±^ mice and cultured as previously described [5]. Briefly, digested VSMCs were cultured in complete medium (DMEM; HyClone, Logan, USA) supplemented with penicillin (100 units/mL; Hyclone), streptomycin (0.1 mg/mL; Hyclone)) and fetal calf serum (10%; Invitrogen, Carlsbad, USA) in humidified 5% CO_2_ atmosphere at 37°C. VSMCs in the experiments were used after three generations. Recombined human platelet-derived growth factor (PDGF)-BB (30 ng/mL; R&D Systems, Minneapolis, USA) were used to treat VSMCs for 8, 12, 24, and 48 h. AG-1296 (MCE, Shanghai, China) was incubated with VSMCs for 48 h. N-acetylcysteine (NAC) (10 nmol/L; Sigma, St Louis, USA) was incubated with VSMCs for 8 h.

### Western blot and immunoprecipitation

Carotid arteries and VSMCs were extracted, lysed with RIPA buffer (Beyotime, Shanghai, China), and then subjected to western blotting analysis as described in our previous research [5]. VSMC extracts were prepared in lysis buffer (50 mmol/L Tris·HCl (pH 7.5), 150 mmol/L NaCl, 0.5% IGEPAL CA-630, 0.5% deoxycholic acid, 0.1% SDS, 1 mmol/L EDTA, 1 mmol/L NaF, 0.1 mmol/L Na_3_VO_4_, 50 μmol/L phenylmethylsulfonyl fluoride, 5 μg/mL leupeptin, 5 μg/mL aprotinin; Solarbio, Beijing, China) for immunoprecipitation assay. Samples were incubated with the primary antibody at 4°C overnight, and immunocomplexes were precipitated after 1 h of incubation with sepharose A/G beads (Santa Cruz, Dallas, USA). Antibodies against PGC-1α (cat ab106814) and GAPDH (cat 2118S) were purchased from Abcam and Cell Signaling Technology, respectively. Antibody against Plin5 was purchased from Novus (cat NB110-60,509).

### Gene expression

Total RNA was extracted from arteries and cells by using Trizol® agent (Life Technologies, Carlsbad, USA) according to the manufacturer’s protocol. The cDNA was synthesized with Bestar™ qPCR RT Kit (DBI Bioscience, Ludwigshafen, Germany). Real-time qRT-PCR was conducted in ABI Prism 7700 Sequence Detector (Applied Biosystems, Carlsbad, USA) by using 2 × SyberGreen mixture (DBI Bioscience). *Gapdh* was utilized as a housekeeping gene. Classic ΔΔCt method was utilized to normalize gene expression. Primers used for each gene were listed as following: *Plin5* (NM_001013706, F, 5’-GAAGTGGGCACAGTGGAGG-3’; R, 5’-AAAGAGTGTTCATAGGCGAGAT-3’), *Calponin* (NM_009922, F, TCTGCACATTTTAACCGAGGTC; R, GCCAGCTTGTTCTTTACTTCAGC) [19], *SM-MHC* (NM_013607, F, AAGCTGCGGCTAGAGGTCA; R, CCCTCCCTTTGATGGCTGAG) [19], *α-SMA* (NM_007392, F, GTCCCAGACATCAGGGAGTAA; R, TCGGATACTTCAGCGTCAGGA) [19], *Gapdh* (AY618199, F, 5’-AGGTCGGTGTGAACGGATTTG-3’; R, 5’-TGTAGACCATGTAGTTGAGGTCA-3’) [15].

### Immunohistochemical staining

Immunohistochemical staining was conducted as previously described [5]. After fixed with 4% paraformaldehyde, the arteries were perfused with saline and embedded in paraffin. The artery sections (4–5 μm) were obtained at 80-μm intervals and then stained with hematoxylin and eosin (H&E; Solarbio). Finally, the areas of intima and media were measured by using Image-Pro Plus software. For immunohistochemical staining, artery sections were incubated with anti-Ki-67 primary antibody (cat 9449 T; Cell Signaling Technology) after being blocked (4°C, overnight). The sections were then incubated with a secondary antibody (Proteintech, Rosemont, USA) and counterstained with Mayer hematoxylin. Five artery sections per sample were collected for staining.

### Immunofluorescent staining

Ki-67 and 4,6-diamino-2-phenyl indole (DAPI) immunofluorescent staining were performed as our previous study described [20]. Primary antibody against Ki-67 were obtained from Cell Signaling Technology (cat 9449 T). For dihydroethidium (DHE; Beyotime) staining, VSMCs were incubated with DHE (5 μmol/L) for 45 minutes (min) at 37°C. After washed with Hank’s balanced salt solution (HBSS; Invitrogen), VSMCs were analyzed under a fluorescence microscope (Olympus, Japan). For immunofluorescent staining of plin5 in artery, frozen section of artery was fixed, blocked, and incubated with rabbit anti-plin5 at 4°C overnight as our previous study described [21]. Then, the section was incubated with a secondary antibody for another 1 h. Images were analyzed by a fluorescence microscope (Olympus).

### Wound healing assay

Wound healing assay was performed as previously described [5]. VSMCs were firstly serum-deprived for 24 h. After treatment of VSMCs, the rates of wound closure were measured via direct microscopic visualization. A reference point in the wound field at the bottom was made to permit photographing of the same spot each time. The remaining cell-free areas were determined at 24 h after injury.

### Transwell assay

The transwell assay was performed as described previously [18]. Briefly, VSMC was incubated in an upper transwell chamber (Millipore, Darmstadt, Germany) and then cultured in a 24-well plate (1 × 10^5^ cells/well) for 8 h. The lower chamber was filled with serum-free DMEM with or without PDGF-BB (30 ng/mL). Non-migrated VSMC was wiped off from the inside of membrane. VSMC on the lower surface was fixed with paraformaldehyde, washed, and then stained for 20 min with 1% crystal violet. Finally, VSMC in four randomly selected fields per well was calculated via a microscope.

### Adenovirus construction

The adenovirus expressing Pgc1α (Ad-Pgc1α), plin5 (Ad-Plin5) and control viruses (Ad-Con) were conducted according to the manufacturer’s instructions (Genechem, Shanghai, China). For transfer in vitro, VSMCs were transfected with adenovirus (3 pfu/cell) for 72 h.

### Statistical analysis

Unpaired Student’s *t*-test was utilized to compare the two independent groups. One-way or two-way analysis of variance (ANOVA) was used to compare means that involve one or two factors, respectively, with appropriate *post hoc* tests. All tests were two-tailed. Data are presented as mean ± S.D. P < 0.05 was considered statistically significant.

## Results

In the current study, we investigated the effect of plin5 on the proliferation and migration of VSMC by utilizing the *Plin5* knockdown mice. We confirmed that plin5 ameliorated proliferation, migration, and oxidative stress in PDGF-BB-challenged VSMC and neointima hyperplasia via interacting with PGC-1α.

### Plin5 is downregulated in injured arteries and proliferating VSMCs

**Figure 1.**
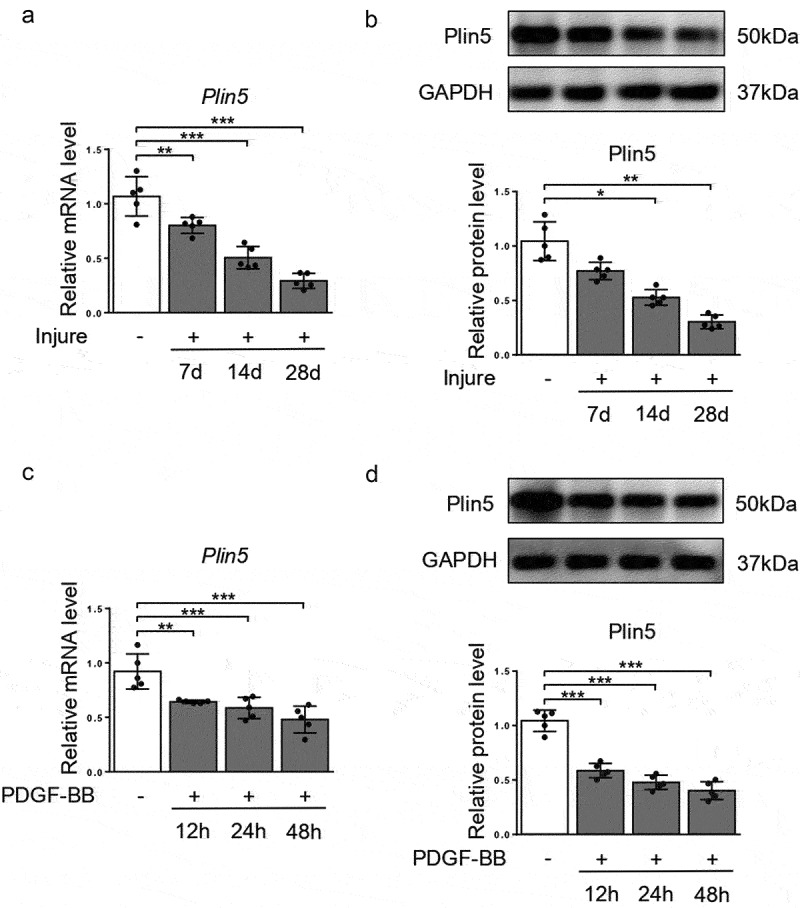
Plin5 is down-regulated in proliferating VSMC and injured artery. The relative mRNA (a) and protein (b) levels of plin5 in common carotid arteries from C57BL/6 J mice at day 7, 14 and 28 after injury were analyzed by qRT-PCR and immunoblotting (n = 5). The relative mRNA (c) and protein (d) levels of plin5 were determined by qRT-PCR and immunoblotting in VSMC after 0, 12, 24 and 48 h of DMSO or PDGF-BB (30 ng/mL) treatment (n = 5). *P < 0.05, **P < 0.01 and ***P < 0.001 denote statistical comparison between the two marked groups, respectively. Data are shown as mean ± S.D.

To investigate the effect of plin5 on vascular intima hyperplasia, we firstly applied a vascular injury model and utilized quantitative real-time polymerase-chain reaction (qRT-PCR) to analyze the mRNA level of *Plin5*. As the result shows, the vascular level of *Plin5* gradually decreased at days 7, 14 and 28 after injury in a time-dependent manner (Figure 1(a)). We also validated the protein expression of plin5 in the injured arteries by western blot. The result shows that vascular protein expression of plin5 significantly declined at days 7, 14 and 28 following mechanical injury (Figure 1(b)). Besides, the fluorescence intensity of plin5 was significantly decreased at day 28 after vascular injury (Supplementary Figure S1). PDGF is secreted from injured sites of vessels and then guides the proliferation and migration of VSMC [22], which then results in neointima hyperplasia. Therefore, we also assessed the expression of plin5 in VSMC after PDGF-BB treatment. Similarly, both mRNA (Figure 1(c)) and protein (Figure 1(d)) levels of plin5 were reduced after PDGF-BB treatment in a dose-dependent manner. However, the decrease in plin5 induced by PDGF-BB was rescued by AG-1296, an inhibitor of PDGF receptor (Supplementary Figure S2), indicating PDGF-BB caused plin5 downregulation in a PDGF receptor-dependent manner. These data suggest that the reduction of plin5 may be associated with neointima hyperplasia and phenotypic switch of VSMC.

### Plin5 knockdown accelerates injury-induced neointima hyperplasia

**Figure 2.**
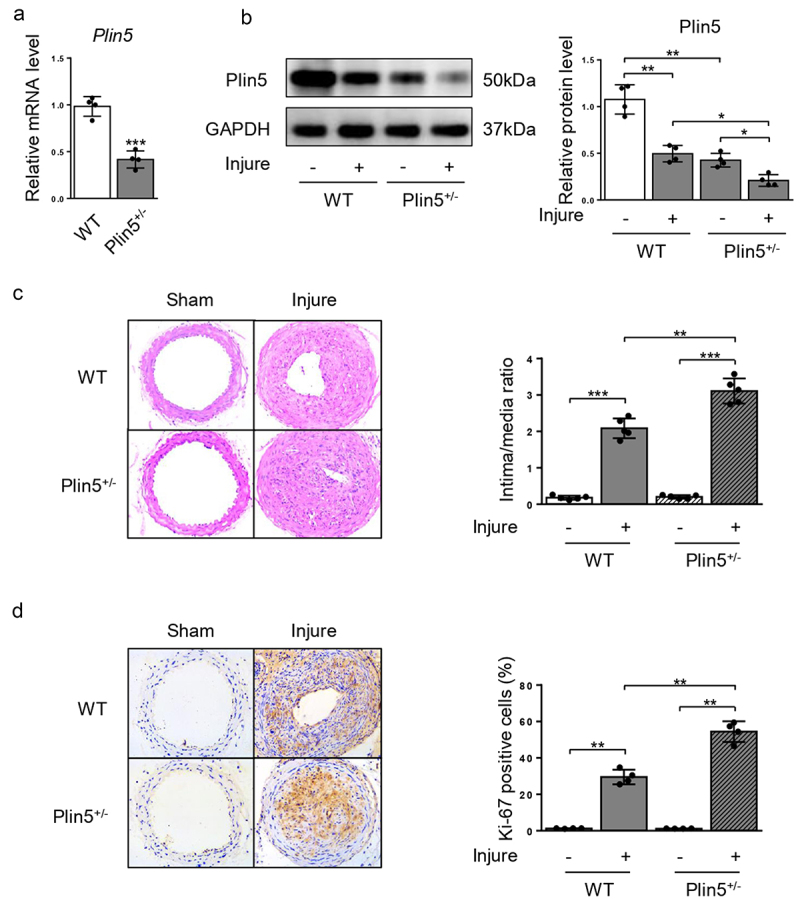
Knockdown of plin5 promotes neointima hyperplasia after vascular injury. The relative mRNA (a) (n = 4) and protein (b) (n = 4) levels of plin5 in common carotid arteries from WT or *Plin5*^±^ mice. (c) Representative H&E staining of carotid arteries from WT or *Plin5*^±^ mice at day 28 after sham operation or wire injury (left) and corresponding quantification for ratio of intima/media (right) were shown (n = 5). Magnification 200 × . (d) Immunohistochemistry staining of Ki-67 (brown) in sections of carotid arteries from WT or *Plin5*^±^ mice at day 28 after sham operation or wire injury (left) and corresponding quantification for Ki-67 positive cells within neointima (right) were shown (n = 4). Magnification 200 × . *P < 0.05, **P < 0.01 and ***P < 0.001 denote statistical comparison between the two marked groups, respectively. Data are shown as mean ± S.D.

Since plin5 was downregulated after wire injury, we further detected whether the decrease in plin5 caused aggravated neointima hyperplasia. Therefore, WT and *Plin5*^±^ mice were subjected to injury of common carotid artery for 4 weeks. Results showed that mRNA (Figure 2(a)) and protein (Figure 2(b)) expression levels of plin5 significantly were reduced in the artery of *Plin5*^±^ mice. At day 28 after injury, the vascular expression of plin5 further declined in *Plin5*^±^ mice compared with that in WT mice (Figure 2(b)). These data suggest *Plin5*^±^ mice exhibited declined vascular plin5 expression in both physiological and pathological conditions. H&E staining of artery showed that injury induced strong intima hyperplasia in both WT and *Plin5*^±^ mice. Furthermore, *Plin5*^±^ mice showed an increased ratio of intima-to-media when compared to WT mice at day 28 following injury (Figure 2(c)). There was no significant difference in the degree of neointima hyperplasia between WT and *Plin5*^±^ mice without wire injury. Ki-67 immunofluorescence staining was used to analyze cell proliferation within the neointima. Injury significantly increased the number of Ki-67-positive cells (proliferating cells) within the vascular neointima in both WT and *Plin5*^±^ mice. More importantly, *Plin5*^±^ mice exhibited larger Ki-67-positive area than WT mice (Figure 2(d)). These data indicate that plin5 knockdown can accelerate neointima hyperplasia and vascular restenosis following injury.

### Plin5 inhibits PDGF-BB-induced VSMC phenotypic transition

**Figure 3.**
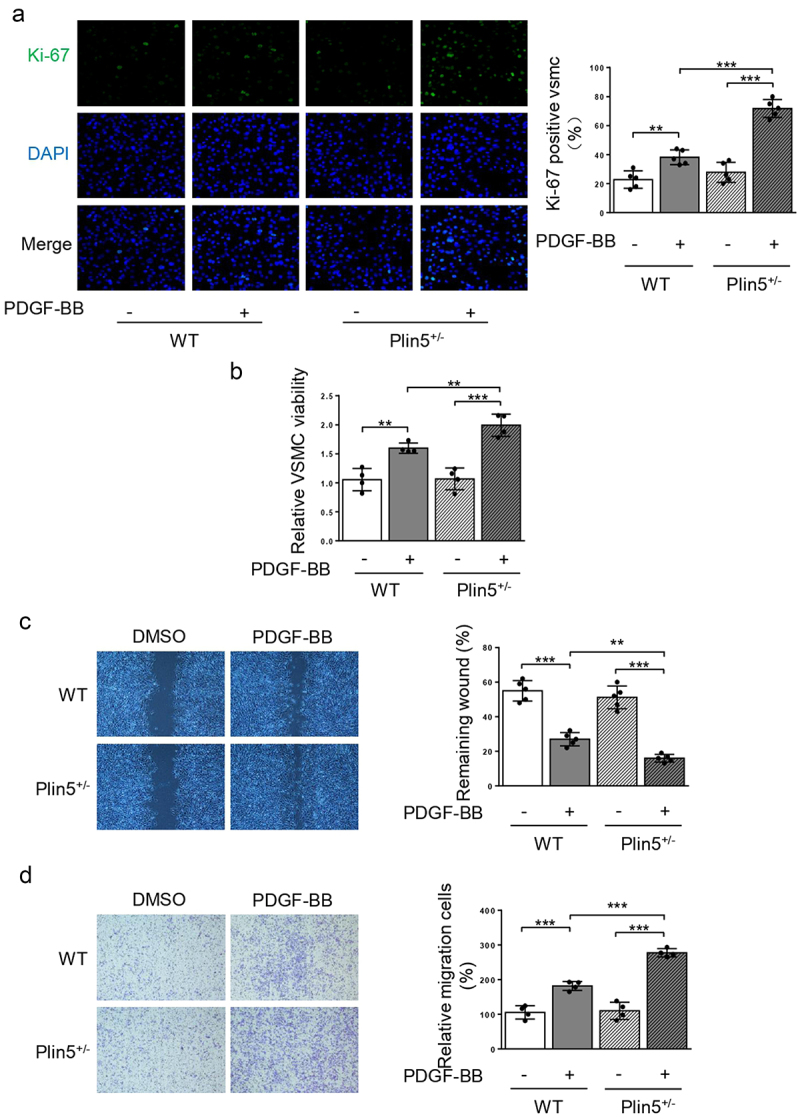
VSMC proliferation and migration in vitro is elevated after plin5 deletion. (a) After DMSO or PDGF-BB (30 ng/mL) treatment for 48 h, VSMC isolated from WT or *Plin5*^±^ mice was stained with Ki-67 (green) and DAPI (blue). Representative images (left) and corresponding quantification of Ki-67 positive VSMC (right) were shown (n = 5). Magnification 400 × . (b) VSMC isolated from WT or *Plin5*^±^ mice was incubated with DMSO or PDGF-BB (30 ng/mL) for 48 h. Then, the absorbance at 450 nm was obtained (n = 4). (c) After 24 h of DMSO or PDGF-BB (30 ng/mL) treatment, migration of VSMC isolated from WT and *Plin5*^±^ mice was measured via wound healing assay. Representative images (left panel) and corresponding quantification of healing rates (right panel) were shown (n = 5). Magnification 100 × . (d) After 8 h of DMSO or PDGF-BB (30 ng/mL) treatment, migration of VSMC isolated from WT and *Plin5*^±^ mice was measured via transwell assay. Representative images (left panel) and corresponding quantification of migration cells (right panel) were shown (n = 4). Magnification 100 × . **P < 0.01 and ***P < 0.001 denote statistical comparison between the two marked groups, respectively. Data are shown as mean ± S.D.

Since the expression of plin5 was reduced following PDGF-BB treatment, we speculated that plin5 might repress the proliferation and migration of VSMC. We firstly utilized Ki-67 immunofluorescence staining to measure cell proliferation. Compared with WT mice, Ki-67-positive area in VSMC was significantly increased in *Plin5*^±^ mice following PDGF-BB incubation (Figure 3(a)), revealing a proliferative effect of plin5 knockdown on VSMC. CCK-8 assay further showed that PDGF-BB could significantly induce the growth of VSMC isolated from both WT and *Plin5*^±^ mice (Figure 3(b)). Moreover, *Plin5*^±^ mice exhibited increased growth of VSMC when compared with WT mice upon PDGF-BB treatment (Figure 3(b)). Overexpression of plin5 by Ad-Plin5 significantly suppressed PDGF-BB-induced increase in number of Ki-67-positive VSMC (Supplementary Figure S3(a)). Therefore, plin5 could repress VSMC proliferation induced by PDGF-BB. We next investigated whether plin5 exerted a certain role in regulating VSMC migration. Our data showed that PDGF-BB treatment caused accelerated wound healing in VSMC isolated from both WT and *Plin5*^±^ mice. Upon incubation with PDGF-BB, plin5 knockdown further promoted VSMC migration. Meanwhile, Knockdown of plin5 did not affect the wound healing rate without PDGF-BB stimulation (Figure 3(c)). Plin5 knockdown also further promoted cell migration upon PDGF-BB treatment by utilizing transwell assay (Figure 3(d)). However, Ad-Plin5 transfection obviously reduced the wound healing rate induced by PDGF-BB (Supplementary Figure S3(b)). To further investigate the role of plin5 in PDGF-BB-induced VSMC phenotypic switch, we also determined the expression levels of VSMC differentiation marker genes such as *Calponin, SM-MHC,* and *α-SMA*. As the result showed, the expression of these genes significantly declined after PDGF-BB for treatment. Transfection of Ad-Plin5 abolished the decrease in the expression levels of *Calponin, SM-MHC,* and *α-SMA* (Supplementary Figure S3(c)). Taken together, these results suggest that plin5 suppresses VSMC phenotypic switch following PDGF-BB treatment.

### Knockdown of plin5 induces proliferation and migration of VSMC via inducing oxidative stress

**Figure 4.**
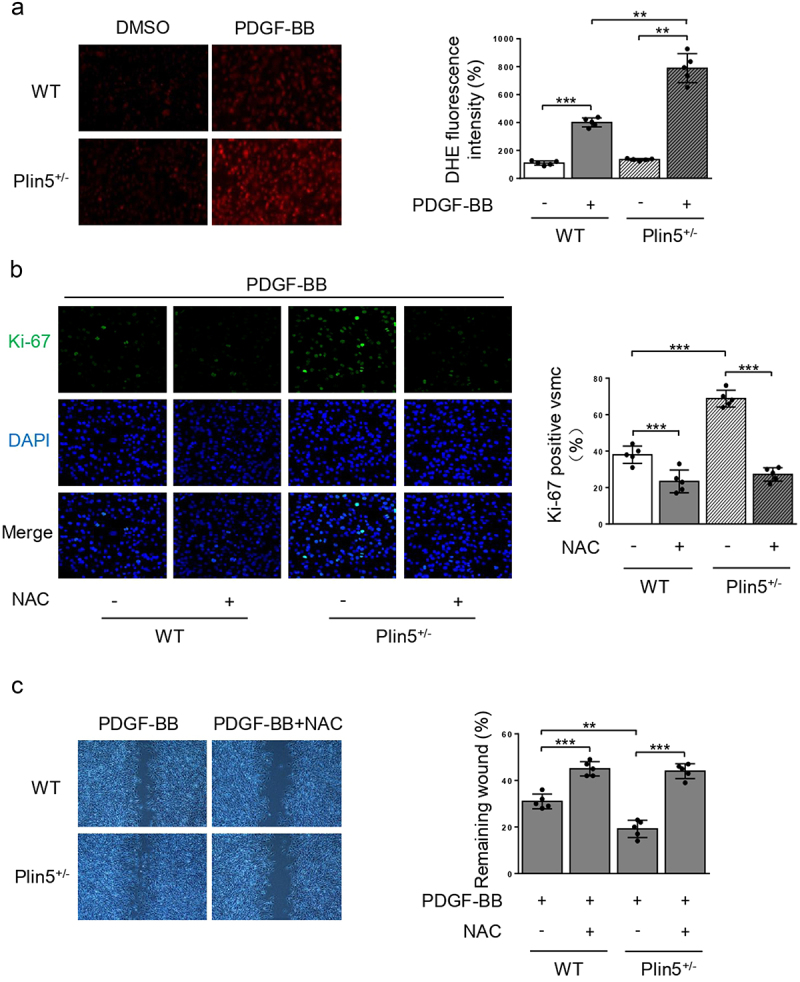
NAC reverses elevated proliferation and migration of VSMC induced by plin5 knockdown. (a) After DMSO or PDGF-BB (30 ng/mL) treatment for 48 h, VSMC isolated from WT or *Plin5*^±^ mice was stained with DHE (red). Representative images (left) and corresponding quantification of DHE fluorescence (right) were shown (n = 5). Magnification 400 × . (b) VSMC isolated from WT or *Plin5*^±^ mice was incubated with DMSO or NAC (10 nmol/L; 8 h) and received 48 h of PDGF-BB (30 ng/mL) treatment. Then, VSMC was stained with Ki-67 (green) and DAPI (blue). Representative images (left) and corresponding quantification of Ki-67 positive VSMC (right) were shown (n = 5). Magnification 400 × . (c) Migration of VSMC was measured via wound healing assay. Representative images (left) and corresponding quantification of healing rates (right) were shown (n = 5). Magnification 100 × . *P < 0.05, **P < 0.01 and ***P < 0.001 denote statistical comparison between the two marked groups, respectively. Data are shown as mean ± S.D.

Excessive ROS production leads to oxidative stress, which contributes to abnormal proliferation and migration of VSMC [16]. Our previous study found that plin5 deficiency caused increased ROS generation in cardiac microvascular endothelial cells [15]. We explored the effect of plin5 knockdown on ROS generation in VSMC. DHE staining showed that ROS content in PDGF-BB group was significantly higher than that of the control group. Compared with WT mice, the elevation of ROS generation following PDGF-BB incubation was more obvious in VSMC isolated from *Plin5*^±^ mice (Figure 4(a)). However, there was no significant difference in ROS generation after plin5 knockdown without PDGF-BB treatment.

Subsequently, we wondered if the elevation of ROS content in VSMC induced by plin5 knockdown was accountable for excessive proliferation and migration. Therefore, we utilized NAC, a potent ROS scavenger, to eliminate plin5 knockdown-induced excessive ROS and analyzed its effect on the proliferation and migration of VSMC. NAC significantly reduced the number of Ki-67 positive VSMC in both WT and *Plin5*^±^ mice. Simultaneously, there was no difference in Ki-67 positive area between WT and *Plin5*^±^ VSMC after NAC application (Figure 4(b)). PDGF-BB-induced VSMC migration was analyzed by scratch wound assay. Consistent with declined VSMC proliferation, NAC also abrogated the promotive effect of plin5 knockdown on migration (Figure 4(c)). These data provided compelling evidence that plin5 inhibition might accelerate the proliferation and migration of VSMC via inducing oxidative stress.

### Plin5 attenuates the proliferation and migration of VSMC by interacting with PGC-1α

**Figure 5.**
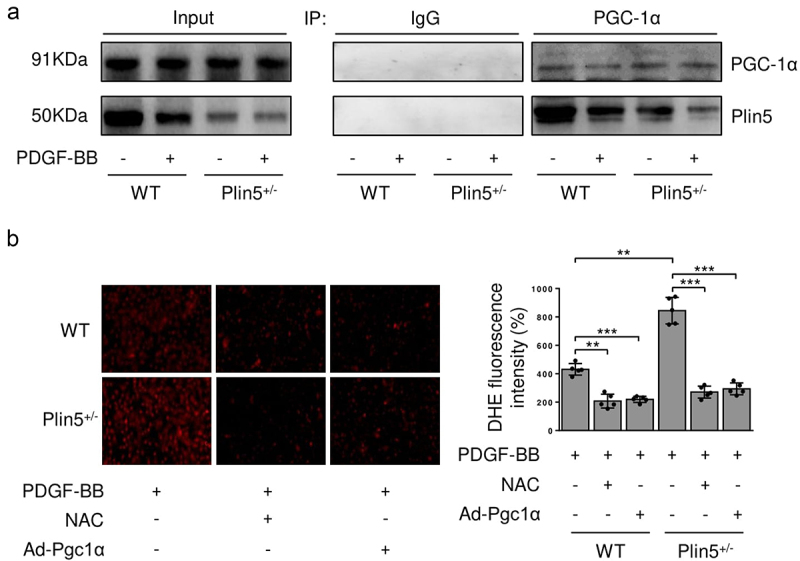
Declined plin5-PGC-1α interaction causes increased ROS level in VSMC. VSMC isolated from WT or *Plin5*^±^ mice after 48 h of DMSO or PDGF-BB (30 ng/mL) treatment was subjected to immunoprecipitation (IP) using anti-PGC-1α antibody or control IgG. (a) Inputs and immunocomplexes were analyzed by immunoblotting. (b) VSMC isolated from WT or *Plin5*^±^ mice was transfected with Ad-Con or Ad-Pgc1α. VSMC was next incubated with DMSO or NAC (10 nmol/L) for 8 h and PDGF-BB (30 ng/mL) for 48 h. Then, VSMC was stained with DHE (red). Representative images (left) and corresponding quantification of DHE fluorescence (right) were shown (n = 5). Magnification 400 × . **P < 0.01 and ***P < 0.001 denote statistical comparison between the two marked groups, respectively. Data are shown as mean ± S.D.

**Figure 6.**
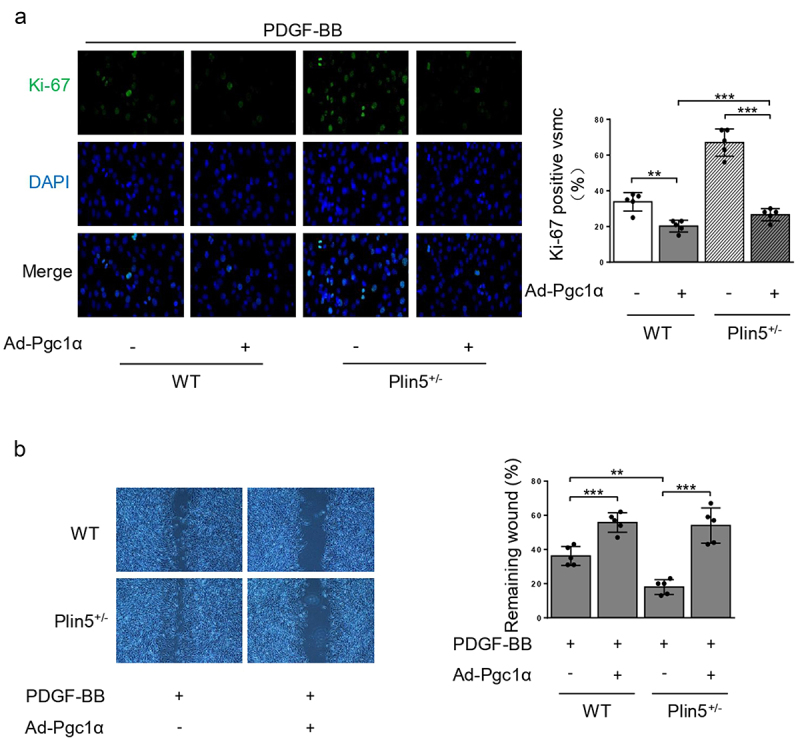
Plin5 knockdown causes increased VSMC proliferation and migration induced by PDGF-BB in a Pgc1α-dependent manner. VSMC isolated from WT or *Plin5*^±^ mice was transfected with Ad-Con or Ad-Pgc1α. VSMC was next incubated with PDGF-BB (30 ng/mL) for 48 h. (a) VSMC was stained with Ki-67 (green) and DAPI (blue). Representative images (left) and corresponding quantification of Ki-67 positive VSMC (right) were shown (n = 5). Magnification 400 × . (b) Migration of VSMC was measured via wound healing assay. Representative images (left) and corresponding quantification of healing rates (right) were shown (n = 5). Magnification 100 × . **P < 0.01 and ***P < 0.001 denote statistical comparison between the two marked groups, respectively. Data are shown as mean ± S.D.

PGC-1α is a critical antioxidant factor and plays an important role in suppressing VSMC proliferation and migration [17]. We investigated whether PGC-1α was involved in ROS generation mediated by plin5 knockdown. PDGF-BB treatment or plin5 knockdown did not affect the protein level of PGC-1α. Immunoprecipitation assay revealed that exposure to PDGF-BB attenuated the interaction of plin5 with PGC-1α. The plin5-PGC-1α interaction further declined in *Plin5*^±^ VSMC (Figure 5(a)). Next, we determined if the declined plin5-PGC-1α interaction was involved in excessive ROS generation. We firstly measure the expression of PGC-1α after transfection of Ad-Pgc1α in VSMC and found that Ad-Pgc1α transfection caused a significant increase in PGC-1α expression (Supplementary Figure S4). Our data showed that NAC and overexpression of PGC-1α by adenovirus transfection had similar ability of eliminating ROS. Moreover, plin5 knockdown failed to elevate the ROS level in VSMC transfected with adenovirus carrying Ad-Pgc1α (Figure 5(b)). These results indicated that excessive ROS generation in *Plin5*^±^ VSMC was caused by attenuated plin5-PGC-1α interaction. We further determined the role of plin5-PGC-1α interaction in the proliferation and migration of VSMC. We found that adenovirus-mediated overexpression of PGC-1α reduced Ki-67 positive VSMC in both WT and *Plin5*^±^ mice. PGC-1α overexpression abrogated the increase of Ki-67 positive VSMC in *Plin5*^±^ mice (Figure 6(a)). Wound healing assay showed that PGC-1α overexpression led to declined VSMC migration in both WT and *Plin5*^±^ mice. After PDGF-BB incubation, Plin5 knockdown failed to further promote migration in VSMC transfected with adenovirus carrying Ad-Pgc1α (Figure 6(b)). Altogether, our data revealed that plin5 knockdown promoted VSMC proliferation and migration via inducing plin5-PGC-1α dissociation and oxidative stress.

## Discussion

Although drug-eluting stents (DES), angioplasty and other percutaneous interventions profoundly improve atherosclerotic cardiovascular diseases, vascular intima hyperplasia, and restenosis still badly influence its clinical prognosis. VSMC phenotypic modulation characterized as excessive proliferation and migration, has been proved to cause vascular neointima hyperplasia after angioplasty [3]. In the current study, plin5 knockdown caused proliferation and migration of VSMC and accelerated intima hyperplasia after vascular injury. Additionally, the binding of plin5 to PGC-1α in VSMC was attenuated after plin5 knockdown. Finally, both overexpression of PGC-1α and antioxidant NAC abolished the promotive effect of plin5 deficiency on the proliferation and migration of VSMC.

Plin5 is originally recognized as a critical factor regulating lipid metabolism in highly oxidative organs such as heart, vessel, and liver [11,23]. By modulating mitochondrial function and lipid metabolism, plin5 also exerts multiple roles in cardiovascular diseases. For example, plin5 deficiency alters lipid metabolism, induces oxidative stress, and causes reduced survival after myocardial ischemia or ischemia/reperfusion [13,24]. Some literature demonstrates that the expression of plin5 is elevated in the atherosclerotic arteries. Plin5 knockout exacerbates severe atherosclerosis in apoe^−/−^ mice via promoting inflammation, lipid accumulation, and oxidative stress [14]. Our present study shows that high glucose and high free fatty acids cause plin5 activation and cardiac microvascular endothelial cell (CMEC) injury [15]. Deficiency or phosphorylation of plin5 promotes CMEC apoptosis and leads to worsened structural incompleteness of capillaries through inducing ROS generation and reducing endothelial nitric oxide synthase expression [15]. In our current study, we found that plin5 expression decreased in the injured arteries. Knockdown of plin5 further accelerated injury-induced vascular intima hyperplasia and stenosis. Thus, plin5 may be a novel target to treat and prevention of vascular restenosis.

As the main component of vascular wall, VSMC exerts an important role in maintaining the integrity of vascular structure and function. When suffering from external stimuli such as mechanical injury, VSMC undergoes a contractile-to-proliferative phenotypic switch, which is characterized by excessive proliferation and migration [25]. This phenotypic switch plays a crucial role in vascular intima hyperplasia. We previously found that ligation, balloon and wire injury could induce vascular intima hyperplasia and stenosis, which were accompanied by abnormal proliferation and migration of VSMC [5,20,21]. In the current study, we also observed declined expression of plin5 in VSMC after PDGF-BB treatment. Moreover, we isolated VSMCs from the thoracic arteries in WT and plin5^±^ mice to investigate the regulatory role of plin5 in VSMC proliferation and migration. Without PDGF-BB treatment, plin5 knockdown showed no obvious effect on VSMC proliferation and migration. Interestingly, PDGF-BB-induced phenotypic switch of VSMC was further enhanced after plin5 ablation but attenuated by plin5 overexpression. We thus speculate that the effects of plin5 deletion on the proliferation and migration of VSMC contribute to injury-induced vascular intima hyperplasia and stenosis.

ROS production is enhanced in VSMC after PDGF-BB stimulus [26] or in injured artery [21]. Although mildly increased ROS is essential for signal transduction, excessive ROS production causes oxidative stress which leads to abnormal proliferation, migration of VSMC and vascular dysfunction [16,27]. Mitochondrial respiratory is the major source of intracellular ROS in VSMC [28]. As a critical regulator of lipid metabolism and mitochondrial function, plin5 attenuates ROS generation in various types of cells, including hepatoma cells [29], cardiomyocytes [24] and endothelial cells [15]. In the current study, plin5 knockdown caused added ROS production, indicating that knock downing plin5 might aggravate oxidative stress in VSMC. When excessive ROS production was abolished by a potent antioxidant, NAC, plin5 ablation failed to further affect proliferation and migration in PDGF-BB-challenged VSMC. These results suggest that plin5 knockdown may induce proliferation and migration of VSMC through promoting excessive ROS production.

Mitochondrial structure and function are modulated by multiple mitochondrial proteins. Our lab previously found that uncoupling protein 2 (UCP2) declined in injured artery and PDGF-BB-challenged VSMC [21]. Decreased expression of UCP2 is responsible for increased ROS generation and enhanced proliferation of VSMC after PDGF-BB treatment. Exposure of VSMC to PDGF-BB causes a significant decrease in the expression of mitofusin 2 (Mfn2), which serves as a critical regulator of mitochondrial fusion and ROS generation [30]. Peroxisome proliferator-activated receptor γ coactivator-1α (PGC-1α) exerts various biological functions, including mitochondrial biogenesis, energy metabolism, fusion, and fission [31,32]. These biological processes are closely related to ROS generation and elimination. Therefore, PGC-1α plays an important role in preventing oxidative stress and VSMC phenotypic transition by modulating its target proteins such as UCP2 and Mfn2 [32,33]. A recent research found that plin5 deficiency upregulated PGC-1α expression in cardiac tissue [34]. Interestingly, PGC-1α content in VSMC was not affected by plin5 ablation in our current study, indicating that oxidative stress induced by plin5 knockdown was not mediated by the alteration of PGC-1α expression. Plin5 is also known to form a complex with PGC-1α in cells [35]. And the plin5/PGC-1α interaction enhances co-activator function of PGC-1α [36]. We observed that plin5/PGC-1α interaction decreased in PDGF-BB-challenged VSMC and the interaction was further attenuated after plin5 ablation. Overexpression of PGC-1α significantly suppressed oxidative stress induced by plin5 knockdown. Moreover, both PGC-1α overexpression and NAC exhibited similar inhibitory effects on ROS production, proliferation, and migration of VSMC after Plin5 knockdown. Taken together, our data suggest that plin5 inhibits PDGF-BB-induced ROS production via binding to PGC-1α and enhancing anti-oxidative activity of PGC-1α.

## Conclusions

In conclusion, our study elucidated the role of plin5 in inhibiting the proliferation and migration of VSMC. More importantly, we demonstrate that plin5 prevents oxidative stress by interacting with PGC-1α. Therefore, we identify plin5 as a novel factor protecting from vascular intima hyperplasia and restenosis following injury.

## Supplementary Material

Supplemental Material
