# Supplementary material for: Plin5 inhibits proliferation and migration of vascular smooth muscle cell through interacting with PGC-1α following vascular injury
Source: Bioengineered. 2022 Apr 26;13(4):10665–78. doi: 10.1080/21655979.2022.2065762 (PMC9161997; doi:10.1080/21655979.2022.2065762)
Supplement: Supplemental Material [file KBIE_A_2065762_SM2048.zip › supplementary/20220314_plin5_Supplementary information.docx]

**Plin5 inhibits proliferation and migration of vascular smooth muscle cell through interacting with PGC-1α following vascular injury**

Xueqing Gan^a,*^, Jiaqi Zhao ^a,*^, Yingmei Chen^a^, Yong Li^b^, Bing Xuan^a^, Min Gu^a^, Feifei Feng^a^, Yongjian Yang^a^, Dachun Yang^a^, Xiongshan Sun^a^

**Supplementary information**

**Supplementary Figure Legends**

Supplementary Figure S1. Pin5 was decreased in injured artery. Representative plin5 immunofluorescent staining (red) of carotid artery from mice at day 28 after sham operation or wire injury were shown. Cell nuclei were counterstained with DAPI (blue). Magnification 200×.

Supplementary Figure S2. PDGF receptor inhibitor abolished PDGF-BB-induced downregulation of plin5 in VSMC. The relative protein level of plin5 was determined by immunoblotting in VSMC after 48 h of DMSO, PDGF-BB (30 ng/mL) or AG-1296 (10 μmol/L) treatment (n=3). **P<0.01 denote statistical comparison between the two marked groups. Data are shown as mean ± S.D.

Supplementary Figure S3. VSMC proliferation and migration in vitro is attenuated after plin5 overexpression. VSMC was transfected with Ad-Con or Ad-Plin5. (a) VSMC treated as above mentioned was incubated with DMSO or PDGF-BB (30 ng/mL) for 48 h and stained with Ki-67 (green) and DAPI (blue). Representative images (upper panel) and corresponding quantification of Ki-67 positive VSMC (lower panel) were shown (n=4). Magnification 400×. (b) VSMC treated as above mentioned was incubated with DMSO or PDGF-BB (30 ng/mL) for 24 h. Migration of VSMC was measured via wound healing assay. Representative images (upper panel) and corresponding quantification of healing rates (lower panel) were shown (n=4). Magnification 100×. (c) VSMC treated as above mentioned was incubated with DMSO or PDGF-BB (30 ng/mL) for 48 h. The relative mRNA of *Calponin*, *SM-MHC* and *α-SMA* were determined by qRT-PCR in VSMC (n=4). *P<0.05, **P<0.01 and ***P<0.001 denote statistical comparison between the two marked groups, respectively. Data are shown as mean ± S.D.

Supplementary Figure S4. Ad-Pgc1α transfection causes increased expression of PGC-1α in VSMC. VSMC was transfected with Ad-Con or Ad-Pgc1α. The relative protein level of PGC-1α was analyzed by immunoblotting (n=4). ***P<0.001 denote statistical comparison between the two marked groups. Data are shown as mean ± S.D.
