# Supplementary figures and images for: Plin5 inhibits proliferation and migration of vascular smooth muscle cell through interacting with PGC-1α following vascular injury
Source: Bioengineered. 2022 Apr 26;13(4):10665–78. doi: 10.1080/21655979.2022.2065762 (PMC9161997; doi:10.1080/21655979.2022.2065762)

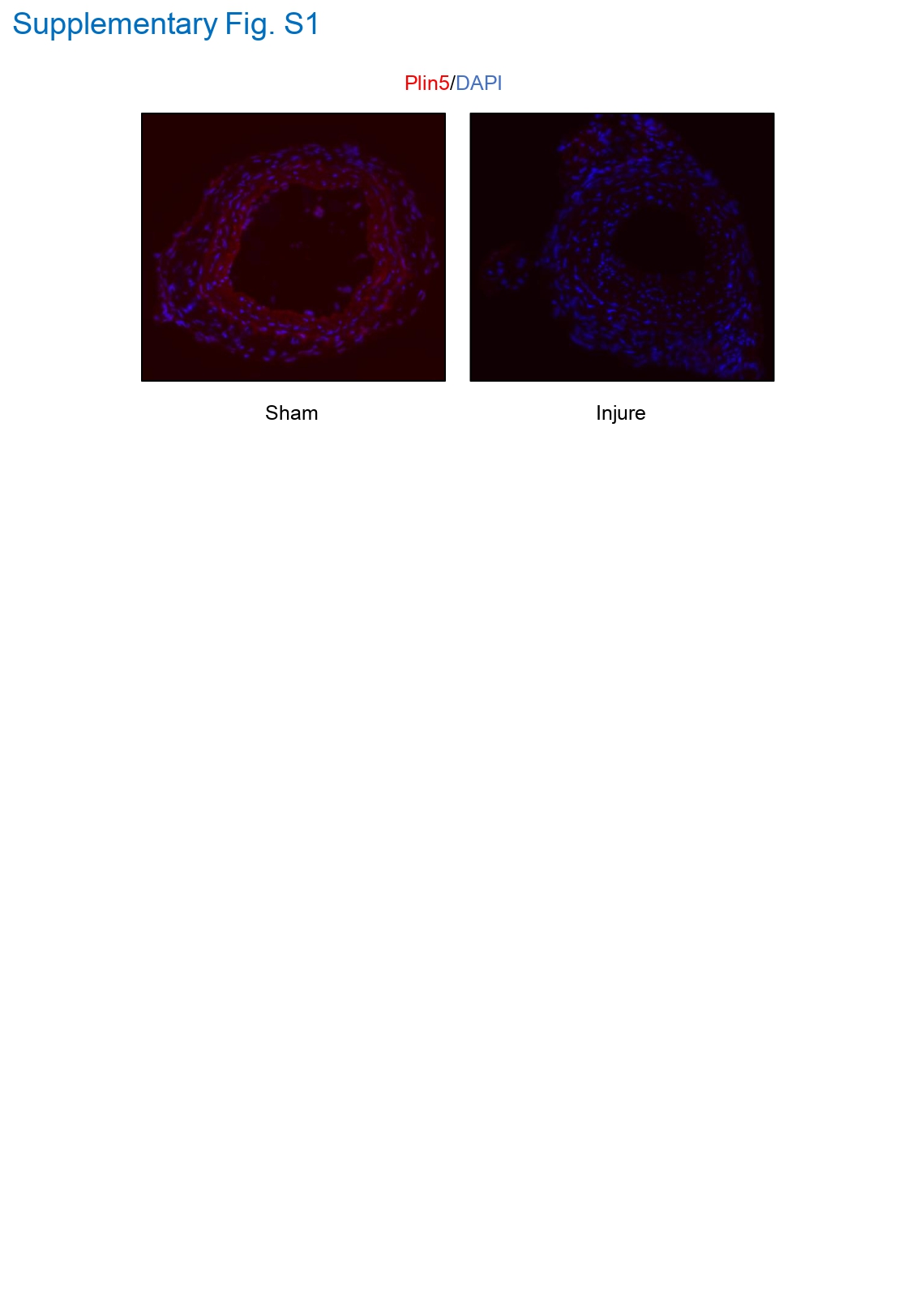

Supplement: Supplemental Material [file KBIE_A_2065762_SM2048.zip › supplementary/Supplementary Figure S1.jpg]

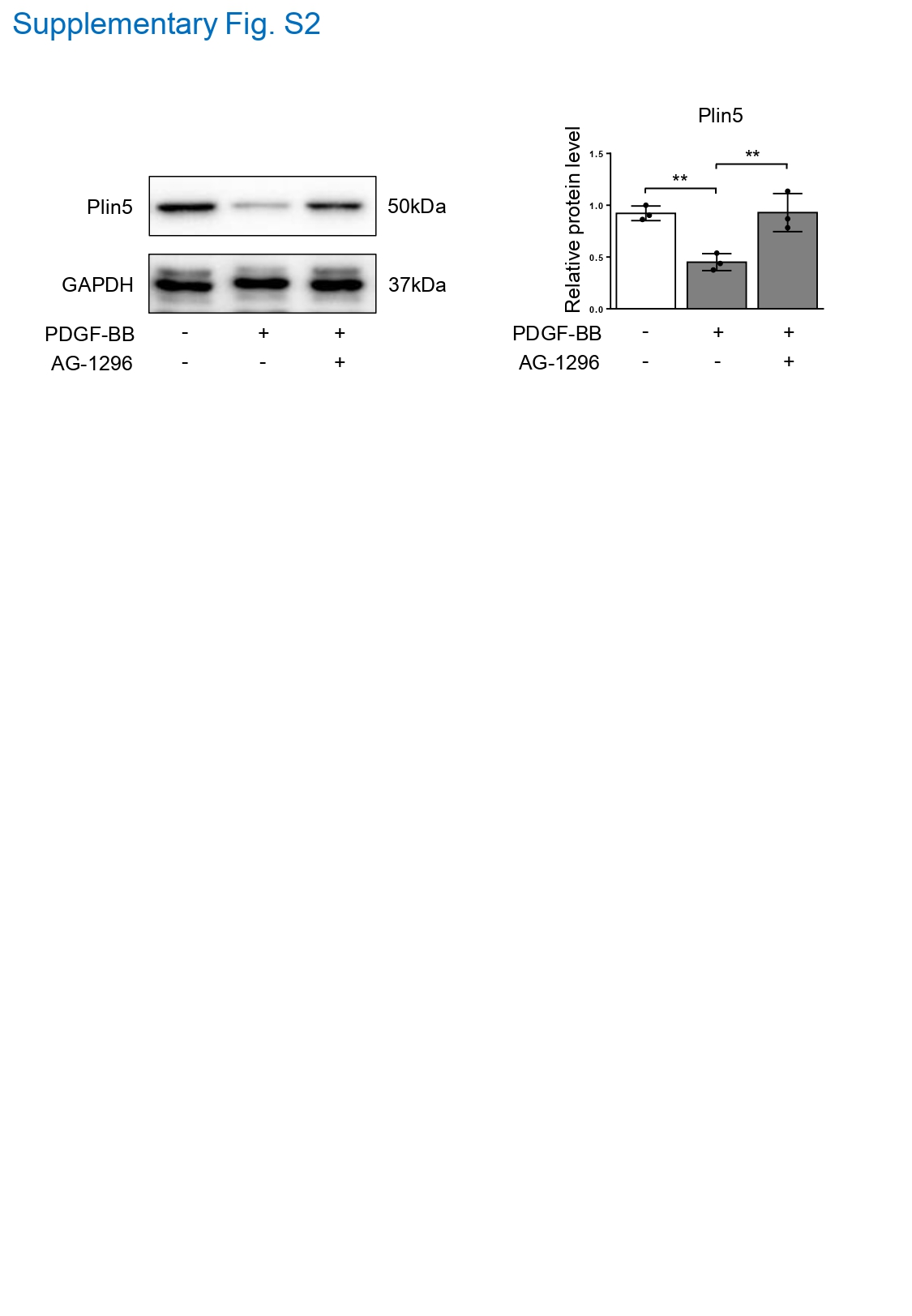

Supplement: Supplemental Material [file KBIE_A_2065762_SM2048.zip › supplementary/Supplementary Figure S2.jpg]

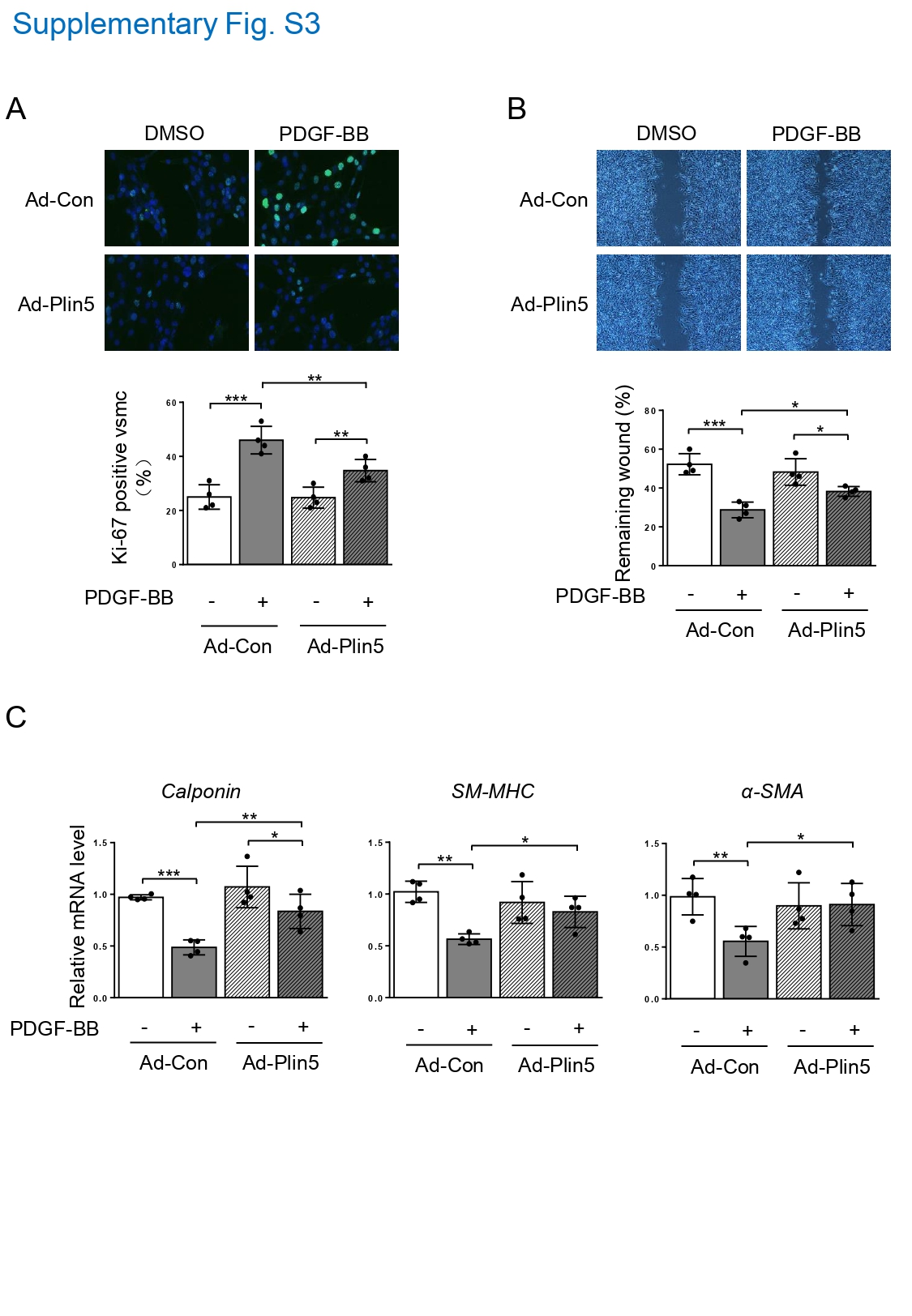

Supplement: Supplemental Material [file KBIE_A_2065762_SM2048.zip › supplementary/Supplementary Figure S3.jpg]

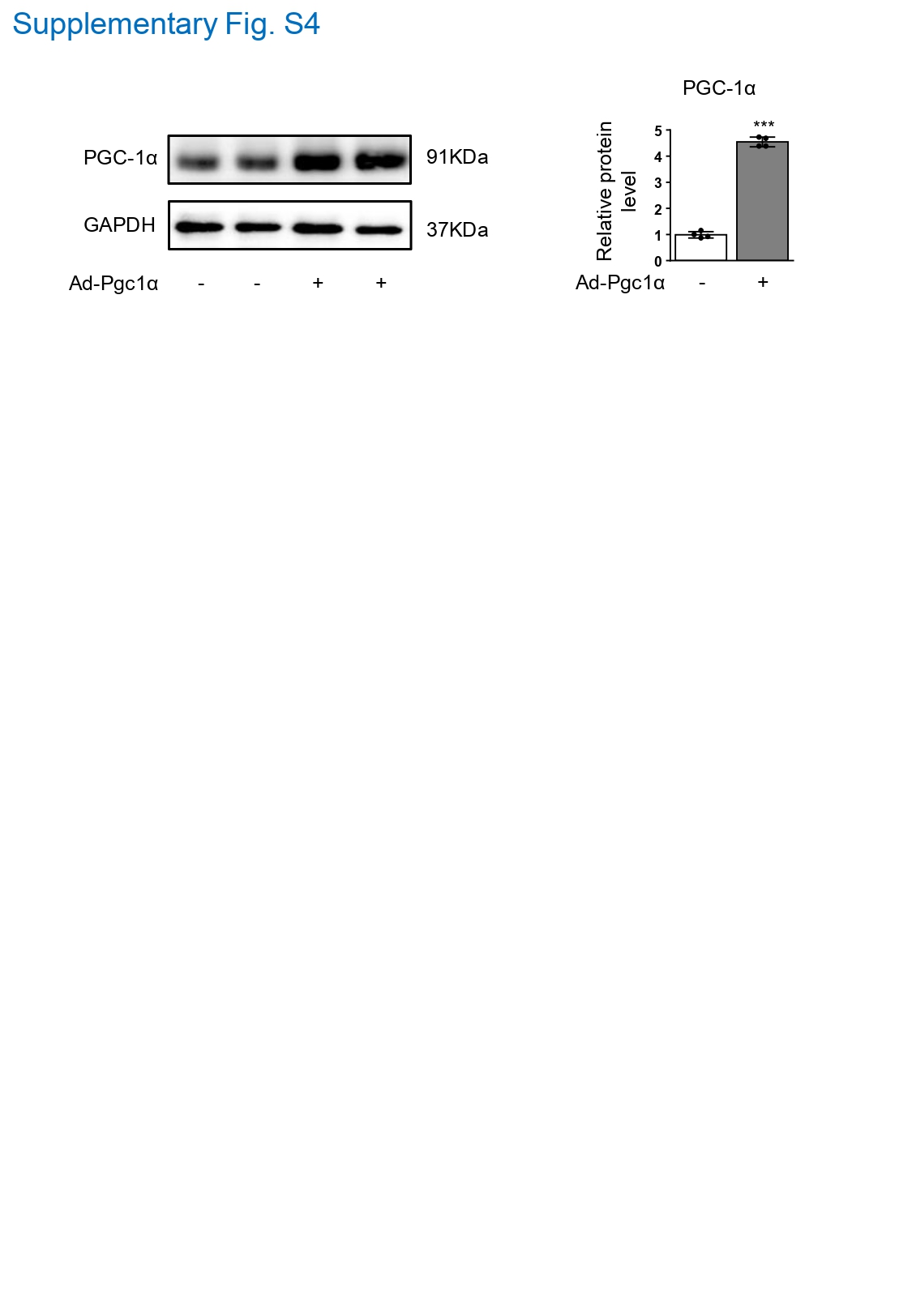

Supplement: Supplemental Material [file KBIE_A_2065762_SM2048.zip › supplementary/Supplementary Figure S4.jpg]
